# Supplementary material for: Risk correlation identification of futures market based on wavelet transform and quantile Granger causality test
Source: PLoS One. 2023 Nov 17;18(11):e0294150. doi: 10.1371/journal.pone.0294150 (PMC10655996; doi:10.1371/journal.pone.0294150)
Supplement: S1 File — (DOCX) [file pone.0294150.s001.docx]

Addendum

A: Risk correlation recognition model of Wavelet transform and quantile Granger Causality test

This paper uses a risk correlation identification model based on wavelet transform and quantile Granger causality test to analyze the dynamic causal relationship and risk correlation degree among four futures yield series (gold, crude oil, soybean and natural gas). The specific steps of the model are as follows:

Discrete wavelet transform is performed on four futures yield sequences, and each sequence is decomposed into approximate components and detailed components, which reflect the long-term trend and short-term fluctuations of the sequence, respectively. ${}$The principle of discrete wavelet transform is to represent a time series in the following form:

$$y_{t}=\sum_{k=-\infty}^{\infty} a_{J}(k)\phi_{J,K}(t)+\sum_{j=1}^{J} \sum_{k=-\infty}^{\infty} d_{J}{(k)\psi}_{j,k}(t)$$

Where $a_{J}(k)$ is the approximation coefficient, $\phi_{J,K}(t)$ is the scale function, $d_{J}(k)$ is the detail coefficient, $\psi_{j,k}(t)$ is the wavelet function, and J is the number of decomposition layers. The scale function and the wavelet function are generated by a given wavelet basis function ψ. In this paper, db4 is used as the wavelet basis function, and the number of decomposition layers is 1.

Quantile Granger causality test is carried out on the approximate components or detail components at different levels of the four futures yield sequences to test whether there is causality between the two sequences at different quantile levels. The principle of the quantile Granger causality test is to compare the sum of squares of residuals of two restricted models and one unrestricted model and construct a statistic to test whether the unrestricted model has a better fitting effect than the restricted model. The restricted and unrestricted models take the following form:

$$y_{t}=\alpha_{0}+\sum_{i=1}^{L} \alpha_{i}y_{t-i}+u_{t}$$

$$y_{t}=\beta_{0}+\sum_{i=1}^{L} \beta_{i}y_{t-i}+\sum_{i=1}^{L} \gamma_{i}x_{t-i}+v_{t}$$

Where $y_{t}$ and $x_{t}$ are two time series, L is the order of lag, and $u_{t}$ and $v_{t}$ are residual terms. If $x_{t}$ has a causal effect on$y_{t}$, then $\gamma_{i}$ is not all zero, and the unrestricted model has a smaller residual sum of squares than the restricted model. The formula for calculating t statistics values is as follows:

$$t_{q}=\frac{(\mathrm{RSS}_{R}-\mathrm{RSS}_{\mathrm{UR}})/L}{\mathrm{RSS}_{\mathrm{UR}}/(T-2L)}$$

Where, $\mathrm{RSS}_{R}$ and $\mathrm{RSS}_{\mathrm{UR}}$are the sum of squares of residuals of the restricted and unrestricted models, respectively, and T is the sample length, $t(L,T-2L;F_{q})$ is the value of the cumulative distribution function of the T-distribution for L and T-2L degrees of freedom at the point $t_{q}$. If ​$\left| t_{q} \right|>1.96$, then the null hypothesis is rejected and$x_{t}$ has a causal effect on $y_{t}$. In this paper, 0.05, 0.5 and 0.95 are used as quantile levels, reflecting the initial state, intermediate state and extreme state respectively. According to the optimal AIC and BIC, the lag order is selected as 1, that is, it is assumed that the influence of one sequence on another sequence only exists in the previous phase.

The quantile regression coefficients and residuals between the four futures yield series are obtained by quantile regression for the approximate or detailed components at different levels. The principle of quantile regression is to minimize the following loss function to obtain a conditional quantile function for a given quantile level q:

$$\min_{\beta}\sum_{t=1}^{T} \rho_{q}(y_{t}-x_{t}^{'}\beta)$$

Where $\rho_{q}(u)=u(q-I(u<0))$ is the test function of the test error u, $I(\cdot)$ is the indicator function, and $\beta$is the quantile regression coefficient. The following quantile regression model is used in this paper:

$$y_{t}=\alpha+\sum_{i=1}^{L} \beta_{i}y_{t-i}+\epsilon_{t}$$

Where $y_{t}$ is a time series, L is the lag order, and $\epsilon_{t}$ is the quantile regression residue.

B: Rolling window analysis function

In this paper, a rolling window analysis function is used to perform the rolling estimation of quantile Granger causality test for two time series, and the change of causality in different time periods is obtained. The formula of this function is expressed as follows:

First, the input parameters are defined, including two time series $y_{t}$ and$x_{t}$, order of delay L, quantile level $q\in(0,1)$, and window size W.

Then, a rolling window function $R(y_{t}{,x}_{t},L,q,W)$ is defined to represent quantile Granger causality test for two time series $y_{t}$ and $x_{t}$ under the lag order L, quantile level q and window size W, and the test statistics $t_{q}(t)$ and P-value $p_{q}(t)$ are obtained. Where $t=L+1,L+2\cdots,T$,T represents the current point in time. The function takes the following form:

$$R(y_{t},tx,L,q,W)=(F_{q}(t),P_{q}(t))=\left[ \frac{(\mathrm{RSS}_{r}-\mathrm{RSS}_{\mathrm{ur}})/L}{\mathrm{RSS}_{\mathrm{ur}}/(W-2L)},1-t(L,W-2L;t_{q}(t)) \right]$$

Where, $\mathrm{RSS}_{r}$and $\mathrm{RSS}_{\mathrm{ur}}$are the sum of squares of residuals of restricted and unrestricted models in the scrolling window, $t(L,W-2L;t_{q}(t)$ is the value of the cumulative distribution function of the t distribution for L and W-2L degrees of freedom at the point $t_{q}(t)$. The restricted and unrestricted models have the following form in the scrolling window:

$$y_{t}=\alpha_{0}+\sum_{i=1}^{L} \alpha_{i}y_{t-i}+u_{t}$$

$$y_{t}=\beta_{0}+\sum_{i=1}^{L} \beta_{i}y_{t-i}+\sum_{i=1}^{L} \gamma_{i}x_{t-i}+v_{t}$$

Where $u_{t}$ and $v_{t}$ are residual terms.

Finally, starting from the L+1 phase, we move forward one phase each time, take w data points as the data in the rolling window, call the rolling window function $R(y_{t}{,x}_{t},L,q,W)$, get the test statistics and p-values, and add them to the test statistics and p-values respectively. Repeat this step until all available data has been used for rolling window analysis. Returns the test statistic and p-value as the result of the quantile Granger causality test under the rolling window.

Finally, from the first phase, move forward one phase at a time, take the data point as the data inside the rolling window, call the rolling window function, get the test statistic and value, and add them to the test statistic and value respectively. $L+1wR(y_{t}{,x}_{t},L,q,W)pp$Repeat this step until all available data has been used for the rolling window analysis. $p$Return the test statistics and values as a result of the quantile Granger causality test under the rolling window. The value of the returned T-statistic is then compared with the critical value of 1.96 to determine whether there is a risk association.
